# Comparison of a hydrophilic and a hydrophobic apodized diffractive multifocal intraocular lens

**DOI:** 10.1007/s10792-013-9727-5

**Published:** 2013-02-05

**Authors:** Jan Willem van der Linden, Ivanka J. van der Meulen, Maarten P. Mourits, Ruth Lapid-Gortzak

**Affiliations:** 1 Department of Ophthalmology, Academic Medical Center, University of Amsterdam, Meibergdreef 9, 1100 AZ Amsterdam, The Netherlands; 2 Retina Total Eye Care, Driebergen, The Netherlands

## Abstract

To compare outcomes between a new design apodized diffractive hydrophilic multifocal intraocular lens (IOL) (Seelens MF; study group), and a well-known apodized diffractive hydrophobic multifocal IOL (SN6AD1; control group). A comparative case series comparing refractive and visual outcomes at distance and near. Patient satisfaction with a validated questionnaire, dysphotopsia and straylight measurement scores were recorded at 3 months post-operatively. The study group comprised 48 eyes and the control group 37 eyes. At 3 months post-operatively the mean uncorrected distance visual acuity (UDVA) was not statistically significant different between the study group and the control group (0.02 ± 0.07 logMAR [SD] vs 0.04 ± 0.09 logMAR). Corrected distance visual acuity (CDVA) was statistically significantly better with the study lens (−0.04 ± 0.05 logMAR vs −0.01 ± 0.04 logMAR (*p* < 0.019). There was no clinical or statistical significant difference at the 40 cm distance (0.09 ± 0.12 logMAR vs 0.08 ± 0.09 logMAR). The study group had statistically significant better uncorrected near acuity at 50 and 60 cm distances (*p* < 0.03 and *p* < 0.007, respectively). In terms of satisfaction the lenses performed equally. Halos were seen less often with the study lens. Straylight, as a parameter for visual quality, was significantly less with the study lens. Conclusion: The Seelens MF performs equally as well as the well-known SN6AD1 for UCDA and CDVA. The Seelens MF performs better at intermediate distance, and seems to allow for better depth of focus, and increased visual quality. More study is needed to corroborate the last finding.

## Introduction

Multifocal intraocular lenses (IOLs), whether diffractive or refractive, have been shown to effectively treat presbyopia [1]. The use of these lenses is limited because of side-effects secondary to the design of the IOLs, high demand in terms of outcome and patient satisfaction that leads to more chair time, and the fact that in most countries patients need to pay more for these lenses [1, 2]. Patients may see halos or have unwanted visual side-effects secondary to the optic design [3]. In an effort to reduce halos seen from refractive and diffractive lenses with a radially symmetric ring design, asymmetric multifocal IOLs have been introduced [4–6]. In the literature, satisfaction is reportedly high. Furthermore, with these lenses visual side-effects are reported between 10–18 %, which is not dissimilar to apodized diffractive IOLs [4–6]. In any type of multifocal IOL in which the image is split into two images that are seen simultaneously, one image will be clear, while the other is hardly perceived or blurred, which in diffractive lenses is usually called the blur circle [3], and is also found in parallel complaints with radially asymmetric lenses [7, 8]. These asymmetric lenses have also been known to cause visual side-effects, which are treated by changing the direction of implantation; with the sectorial addition upward, or by inserting a capsular tension ring to reduce tilt and decentration [9]. Reading comfort depends on the addition in the lens. In lenses were the addition is 3.75 D at the IOL plane, the intermediate vision will suffer more on account of the very near addition at 33 cm. However, introduction of lenses with a +3.0 D addition in the IOL plane have a maximal near vision at 42 cm. Depending on the type of multifocal IOL and the profile of the refractive/diffractive surface, more or less intermediate vision is gained or lost [10]. The Seelens MF hydrophilic IOL is a multifocal diffractive apodized IOL in which the apodization distances were adjusted in order to produce two foci, one for near and one for far, and to reduce the often seen side-effect of seeing halos from the diffractive rings on the IOL optic, while maintaining a balance in the light distribution between distance and near. The IOL is pupil dependent, with distance dominance under mesopic conditions. The profile was designed as such to maximize near vision and optimize distance vision. The basic design is that of an apodized diffractive lens [8].

Here we report the results of a comparative study of a new apodized diffractive multifocal IOL of hydrophilic material compared to a well-known apodized diffractive hydrophobic IOL in terms of visual, refractive, straylight, patient satisfaction, and side-effects.

## Methods

Two consecutive groups of patients were prospectively compared. The patients had either a Seelens MF or a SN6AD lens implanted. The indication for surgery was either cataract or refractive lens exchange. The tenets of the declaration of Helsinki were adhered to. The guidelines of the Dutch Society of Refractive Surgeons were followed. All patients provided informed consent. Exclusion criteria were patients with ocular disease other than cataract (e.g., cataract, macular disease, dry eye syndrome), corneal astigmatism over 1.25 D, systemic disease such as diabetes with or without retinopathy and an American Society of Anesthesiologists classification of III and higher and systemic disease such as diabetes with or without retinopathy. Both groups of patients had multifocal diffractive IOLs implanted for cataracts or for refractive purposes. There was no randomization. All consecutive cases of these types of lenses between January 1st 2011 and December 31st 2011 were included.

## IOL selection and characteristics

After extensive counselling and at the patient’s and the surgeon’s discretion a lens was chosen. Patients were told that both lenses were diffractive, that one type is a lens with which there is extensive experience and good results, and the other lens is a newer type in which there is less clinical data available, but improvements have been made to the apodized diffractive rings, that the material is free of glistening, and that the 360 degree round edge may provide extra protection against posterior capsular opacification.

The Seelens MF (Hanita Lenses, Israel) is a hydrophilic apodized diffractive lens with an overall diameter of 13 mm diameter, made of Benz26 material. The 11 apodized diffractive rings extend to a diameter of 4 mm on the 6 mm biconvex optic. The lens has two C-loop haptics with 5 degree angulation and 360-degree sharp-edged optic, separating this from the haptics, to prevent posterior capsular opacification. The lens is injected with SoftJect 1.8 injector and cartridge (Hanita Lenses). The lens has a +3 D addition in the IOL plane.

The SN6AD1 (Alcon, Fort Worth, USA) is a well-known apodized diffractive hydrophobic lens of Acrysof material (Alcon), with nine rings extending 3.6 mm onto the 6 mm biconvex optic. The lens has a sharp edge extending on the whole IOL optic and haptic surface, with no edge between the haptic and the optic. The lens is not angulated. The near addition is +3.0 D in the IOL plane.

## Surgical technique

All surgeries were performed under local anesthesia with an oral sedative (Oxazepam 10 mg) administered 20 min prior to the procedure. The pupil was dilated with 1.0 % cyclopentolate instilled three times with 5 min apart and intracamerally with 1:10,000 phenylephrine in balanced salt solution. Anesthesia was achieved with 1.0 % oxubupivocaine and 1.0 % tetracaine drops and a 0.5 mL subconjunctival injection of 2 % lidocaine.

The surgery was performed using standard phacoemulsification technique through a 2.2-mm incision at the 12 o’clock position. The IOLs were implanted with the injectors supplied by the respective manufacturers. Target refraction was emmetropia in all cases. In the Seelens MF the SRK-T formula was used, and for the SN6AD1 the Haigis formula with optimized constants was used.

## Outcome measures and statistical analysis

The primary outcome measure is the uncorrected/corrected distance visual acuity between the study and the control group. The secondary outcome measure is patient satisfaction, in which we expect the newer lens to have fewer complaints with regard to halos. Pre-operative assessment included a complete refractive and ophthalmologic examination, topography and pupillometry with the Orbscan (Technolas, Germany) and biometry with IOLMaster (Zeiss, Germany). Pre- and post-operatively straylight was measured with the C-Quant straylight meter (Oculus Germany). Post-operative incidence of halos was assessed at 3 months.

Full refractive and ophthalmic examination with visual acuity and refraction was performed at 3 months and compared to the pre-operative parameters. Uncorrected distance visual acuity (UDVA), corrected distance visual acuity (CDVA), uncorrected near visual acuity (UNVA) and corrected near visual acuity (CNVA) was assessed pre- and post-operatively. The achieved refraction was calculated in spherical equivalent (SE) refraction and compared between the groups. The change in straylight was measured and analyzed. Post-operative rates of outcome between ± 0.5 D and ± 1.0 D were calculated. Near visual acuity was measured post-operatively at 3 months at distances between 30 and 70 cm with 10-cm intervals and compared between the study group and the control group. Complications were registered and analyzed.

Statistical analysis was performed using PAWS Statistics software (version 18.0 SPSS, Inc). When applicable, nonparametric analysis was performed using the Student *t*, Chi squared, and Pearson tests.

## Results

### Demographics

The study group comprised 48 eyes of 25 patients. The control group had 37 eyes of 20 patients. Table 1 shows the patient demographic data. The groups were well matched in terms of age, indications for surgery, and CVDA. In terms of refraction there was a statistically significant difference, but the axial lengths and anterior chamber depths were well matched. The pre-operative pupil diameters were well matched.

**Table 1:** Pre-operative between-group comparison of patient demographics

Demographic data	Study group	Control group	*p* value
Eyes	48	37	−
Female sex (%)	7 (28)	9 (45)	0.18
Mean age (years) ± SD	57.4 ± 2.81	59.6 ± 7.49	0.14
Mean CDVA (logMAR) ± SD	0.10 ± 0.62	0.09 ± 0.13	0.20
Indication for surgery			0.16
Cataract (%)	28 (58)	27 (73)	
RLE (%)	20 (42)	10 (27)	
Sphere (D)			
Mean ± SD	1.14 ± 1.59	0.31 ± 3.12	0.051
Range	−3.5 D to +5.75 D	−6.5 D to +5.25 D	
Cylinder (D)			
Mean ± SD	−0.45 ± 0.38	−0.67 ± 0.32	0.009
Range	0 to −1.25	−0.25 to −1.50	
Spherical equivalent (D)			
Mean ± SD	1.19 ± 1.68	−0.02 ± 3.06	0.035
Range	−3.88 to +5.13	−6.88 to +5.00	
Axial length			
mm ± SD	23.47 ± 1.56	23.84 ± .78	0.30
Range	22.17–25.54	21.01–27.45	
Anterior chamber depth			
mm ± SD	3.33 ± 0.12	3.24 ± 0.43	0.34
Range	2.61–3.93	2.70–4.56	
Pre-operative pupil diameter			
mm ± SD	3.39 ± 0.21	3.46 ± 0.85	0.49
Range	2–4.1	2.3–4.6	

*CDVA* corrected distance visual acuity, *RLE* refractive lens exchange, *SE* spherical equivalent, *NS* not significant

### Refractive and visual outcomes

Table 2 shows the pre- to post-operative change in refractive parameters. The post-operative SE outcomes were very close to emmetropia. The pre- to post-operative differences were statistically significant for the sphere and SE in the study group. The refractive changes in the control group were not statistically significant. Table 3 shows the between-group comparison of the post-operative outcomes. In terms of spherical outcomes and SE the study and control groups had very similar outcomes. The difference in the cylindrical outcome is statistically significant better with the study lens. In the study group 44 eyes (92 %) were within 0.5 D of emmetropia, and 47 eyes (98 %) were within 1.0 D of emmetropia. In the control group 35 eyes (95 %) were within 0.5 D of emmetropia, and all eyes (100 %) were within 1 D of emmetropia.

**Table 2:** Change in sphere, cylinder and spherocylindrical equivalent pre-operatively to post-operatively

Group/parameter	Pre-operative	Post-operative	*p* value
	Mean (D) ± SD	Mean (D) ± SD	
Study			
Sphere	1.41 ± 1.59	0.23 ± 0.42	<0.0001
Cylinder	−0.45 ± 0.38	−0.41 ± 0.39	0.24
SE	+1.18 ± 1.68	0.03 ± 0.40	<0.0001
Control			
Sphere	0.31 ± 3.12	0.29 ± 0.25	0.65
Cylinder	−0.67 ± 0.32	−0.63 ± 0.42	0.91
SE	−0.02 ± 3.06	0.07 ± 0.16	0.81

*SE* spherical equivalent

**Table 3:** Post-operative comparison between the study and the control groups

Parameter	Study group	Control group	*p* value
Sphere (D)			
Mean ± SD	0.23 ± 0.42	0.29 ± 0.25	0.43
Range	+1.75, −0.75	+0.75, −0.25	
Cylinder (D)			
Mean ± SD	−0.41 ± 0.39	−0.63 ± 0.42	0,041
Range	0, −1.50	0, −1.50	
SE (D)			
Mean ± SD	0.03 ± 0.40	0.07 ± 0.16	0.54
Range	+1.50, −0.75	+0.38, −0.25	

**Fig. 1:**
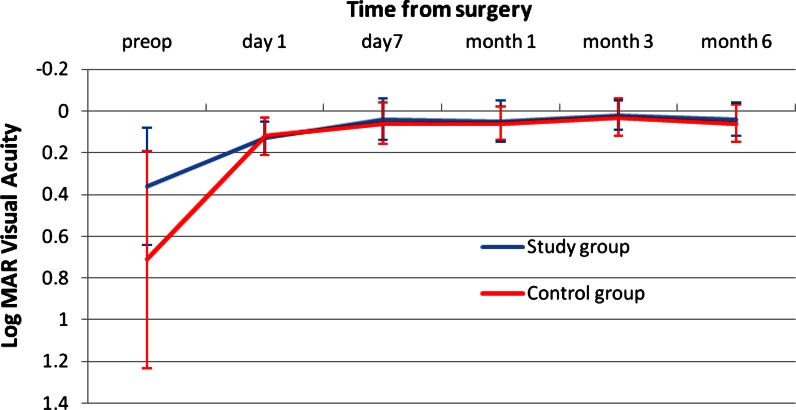
Mean uncorrected visual acuity up to 6 months after surgery. At all time-points measured post-operatively the study group and the control group performed equally in terms of uncorrected distance visual acuity and were not statistically significantly different

**Fig. 2:**
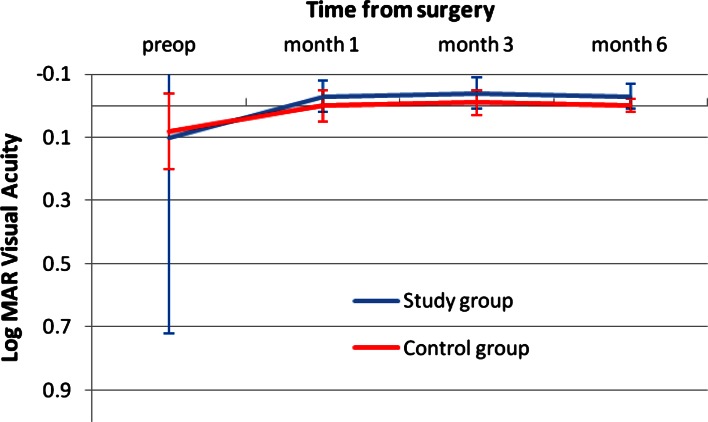
Comparison of the post-operative corrected distance acuity up to 6 months. The difference between the groups is small but statistically significant in favor of the study group (*p* < 0.019)

**Fig. 3:**
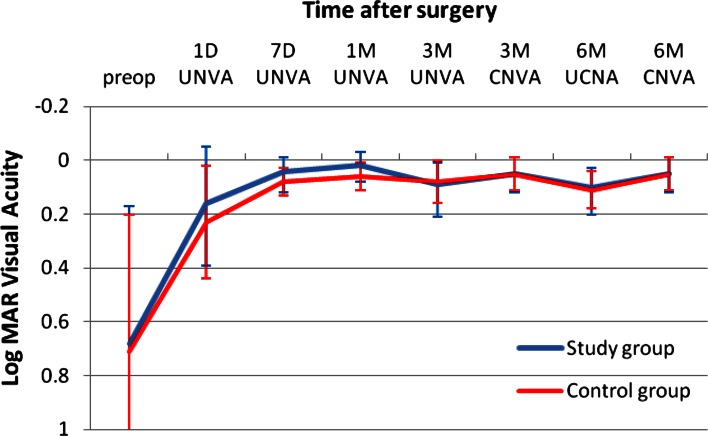
UNVA at 40 cm at different time-points in the follow-up period. The study group and the control group perform equally well. There were no clinical or statistically significant differences between the groups

**Fig. 4:**
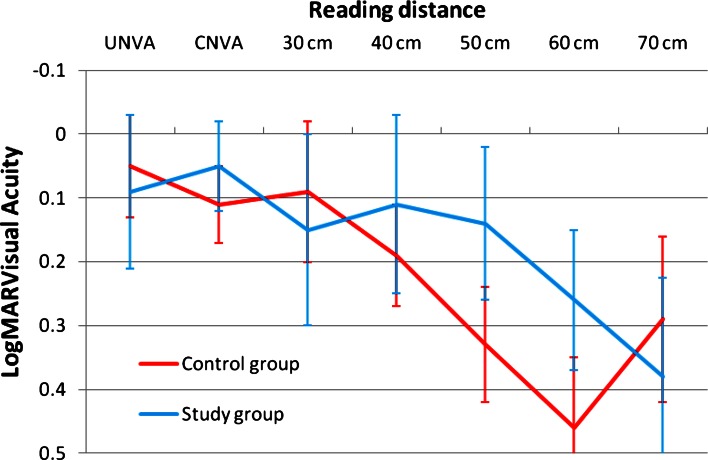
Difference in near acuity at different distances with or without correction. There is no clinical or statistical difference for the 30 and 40 cm distance between the study and control groups. However, there is a clinical and statistically significant better reading at 50 and 60 cm for the study group (*p* < 0.03 at 50 cm and *p* < 0.007 at 60 cm)

Figure 1 shows the change in UCDA post-operatively and the difference between the study and the control group. The study group was slightly better than the control group with a mean UDVA of 0.02 ± 0.07 logMAR versus 0.04 ± 0.09 in the control group. This was not clinically or statistically significant. Figure 2 shows the outcomes of CDVA for the study group and the control group. The difference at 3 months is in favor of the study group with a mean CDVA of logMAR −0.04 ± 0.05 in the study group versus logMAR −0.01 ± 0.04 in the control group (*p* < 0.019). Figure 3 shows the UNVA at 40 cm throughout the first 3 months. The results are comparable for the study and the control groups and were maintained during the 6 months of follow-up. Figure 4 shows the UNVA and CNVA at 40 cm and the differences in UNVA at 10-cm incremental intervals. The study group has a mean logMAR of 0.09 ± 0.12 UNVA at 40 cm and the control group has a mean logMAR of 0.08 ± 0.08 UNVA at 40 cm. This was not clinically or statistically significant. However, there was a clinical and statistical significant difference at the 50 and 60 cm distances where the study group performed better than the control group (*p* < 0.03 for 50 cm and *p* < 0.007 for 60 cm).

### Straylight

Straylight at 3 months changed from a mean log S of 1.276 ± 0.078 in the Seelens MF group to 1.077 ± 0.237 (*p* < 0.0001). In the SN6AD1 group straylight reduced less from 1.243 ± 0.594 pre-operatively to 1.189 ± 0.0194 post-operatively (*p* < 0.25). The mean difference between the study and the control groups post-operatively was a −0.12 log S in favor of the study group (*p* < 0.002).

### Halos

Halos were reported at 3 months in three (12 %) patients in the study group and five (28 %) patients in the control group. This difference did not reach statistical significance, even though there is a clinical significance.

### Complications

In one eye in the study group a decentered lens with capsular phimosis was operatively decentered with an UCDA of 0.16 logMAR and a CDVA of 0 three months after the intervention. In the control group a case of capsular phimosis that needed surgery had a UCDA of 0.2 post-operatively and a CDVA of 0. After these second interventions no further problems were encountered in these patients.

### Satisfaction

Overall, 24 (96 %) patients in the study group were satisfied with the multifocal IOLs. One patient was dissatisfied, and this was because of a residual refraction of S + 0.25C − 0.50 × 125. The uncorrected vision in this eye was 0.06 logMAR while in the other eye it was −0.06 with a plano refraction. In the control group 19 (95 %) patients were satisfied with the surgery and the effect on vision. The one patient who was not satisfied had a UDVA of logMAR 0.1 and a CDVA of logMAR 0, with a refraction of S 0 C − 0.25 × 110. The other eye had a plano refraction and UDVA of −0.08. There was no clinical or statistical difference in satisfaction between the study and the control groups.

## Discussion

In the past decade the use of multifocal diffractive and refractive lenses has developed tremendously [10]. The surgeon has a wide range of choice in terms of IOL materials, refractive of diffractive profiles, and addition profiles, while many patient-related factors play an important role [10]. In this study we have shown that the latest addition in terms of diffractive apodized multifocal lenses, i.e., a lens of hydrophilic material, compares very well to a well-known and widely used apodized diffractive lens of hydrophobic material [11].

In terms of CDVA the study lens (Seelens MF) performed clinically slightly better than the control lens (SN6AD1), but this difference was statistically significant. In terms of UDVA the two lenses are on par. For the UNVA the lenses show a different functional profile. The reading at the 30 and 40 cm distance is excellent with both lenses, and statistically there is no difference. However, in the study group the UNVA at distances between 50 and 60 cm was statistically better than in the control group. Pre-operative pupil size could not account for this, as the groups were well matched. Post-operative pupil sizes are expected to react similarly as no complications relating to iris integrity occurred. However, corneal higher order aberrations were not measured or taken into account. One of the reasons for this is possibly the change in the profile and the apodization of the newer IOL, which allows for more depth of focus for the near vision focus [12].

The mean refractive outcomes compare very well between the groups. We see a larger spread of the achieved refraction with the Seelens MF. In the Seelens MF, because of its novelty, the optimized a_0_, a_1,_ and a_2_ constants for the Haigis formula were not yet available at the time of surgery, and as a result lens calculations had to be made with a formula that does not take into account the effective lens position with constants. We expect that with more experience and elucidation of the constants the results will improve, and that mainly the prediction of the post-operative anterior chamber depth will be the main source of IOL calculation error [13].

Straylight is a reliable and repeatable measure of visual quality [14–16]. The measurements at 3 months showed a clinical and statistical significant difference in favor of the Seelens MF. A significant decrease in straylight was found post-operatively in the study group (−0.20 log S, *p* < 0.0001), and significantly less straylight in the study group compared to the control group post-operatively (−0.12, *p* < 0.002). The adjustment of the apodized diffractive profile possibly allows for more light to reach the retina without disturbance, and less forward scatter. The mechanism directed at reducing post-operative halos from the diffractive profile also improved visual quality, as demonstrated by the reduction in straylight compared to the control group. The effect of diffractive multifocal IOLs on straylight is small. In two studies by the same group, Cerviño et al. found that there was no difference in straylight between eyes implanted with a monofocal IOL versus a group implanted with the SN6AD3 diffractive apodized IOL (ReSTOR, Alcon) [17, 18]. In these studies there was no relationship between subjective complaints of halos and glare and objectively measured straylight [17, 18]. de Vries et al. found a small but significant lower straylight in monofocal lenses, and concluded this was caused by the diffractive pattern of the multifocal IOL [19]. Ehmer et al. found that refractive multifocal IOLs have less straylight, but more halos and subjective complaints than diffractive or segment addition IOLs; however, each study group consisted of only 10 eyes [7]. Glistenings as a source of increased straylight in the hydrophobic (control) group versus the hydrophilic (study) group is probably not the cause for the difference in straylight. One reason is because glistenings develop over time, and here the cut-off point was 3 months, and the other reason is because glistenings behave like a localized effect, comparable, for example, to defects caused by pitting of the IOL when performing a Nd-YAG-laser capsulotomy; the defects are not large enough to be detected by straylight measurements [20]. Since the outcomes of straylight in multifocal IOLs in the literature are mixed, this topic needs attention in future research.

Halos play a role in visual quality after surgery in all diffractive and refractive IOLs with a symmetrical concentric design with rings. We found at 3 months that the Seelens MF group had less halos (in terms of incidence 12 % versus 28 %); however, this difference was not statistically significant, even though there was a trend to significance (*p* < 0.12). We think that the lack of significance could be solved by enlarging the sample sizes.

Patient satisfaction was high in both groups. Interestingly, there seems to be no relationship between the actual observed and objectivised outcome and patient dissatisfaction. Two patients, one in each group, were not satisfied with the multifocal IOL. In both instances the patients had a relatively good refractive outcome but with some asymmetry, with one eye having excellent UCDA and the other a minor residual refractive error. Both patients did not opt for a lens exchange, and the residual error was deemed to be too small for corneal laser enhancement. We now know after further follow-up that both patients adjusted to the situation.

In comparison with historical data, we see that satisfaction is as high as we expect it to be with apodized diffractive multifocal IOLs. The incidence of halos in a previous study was approximately 18.18 %, while in our study it was 28 % [5]. We think this might be related to our sample size. If the difference between the halos is real, a larger sample needs to be examined in order to determine whether the improvements and adjustment of the apodized diffractive profile of the IOL reduce halos. Clinically this already seems to be the case.

## Conclusion

The Seelens MF performs well compared to a well-known multifocal apodized IOL, the SN6AD1. The lens material and design of the Seelens MF show a clinical and statistically significantly improvement in straylight and quality of vision. Clinically the incidence of halos was less in the study group; however, this was statistically not significant. Near acuity was comparable in both groups, with a clinically and statistically significant advantage for the Seelens MF at the 50 − 60 cm distances. The Benz26 material makes the Seelens MF free of glistenings, but the SN6AD1 is a lens that has been used more often with excellent results, and excellent possibilities of accurate IOL calculation.
